# Infinite order decompositions of C*-algebras

**DOI:** 10.1186/s40064-016-3468-7

**Published:** 2016-10-21

**Authors:** Arzikulov Farhodjon Nematjonovich

**Affiliations:** Faculty of Mathematics, Andizhan State University, Andizhan, Uzbekistan

**Keywords:** C*-algebra, Peirce decomposition, Von Neumann algebra, 54C40, 14E20

## Abstract

The present paper is devoted to infinite order decompositions of C*-algebras. It is proved that an infinite order decomposition (IOD) of a C*-algebra forms the complexification of an order unit space, and, if the C*-algebra is monotone complete (not necessarily weakly closed) then its IOD is also monotone complete ordered vector space. Also it is established that an IOD of a C*-algebra is a C*-algebra if and only if this C*-algebra is a von Neumann algebra. As a summary we obtain that the norm of an infinite dimensional matrix is equal to the supremum of norms of all finite dimensional main diagonal submatrices of this matrix and an infinite dimensional matrix is positive if and only if all finite dimensional main diagonal submatrices of this matrix are positive.

## Background

The present paper is devoted to the notion of infinite order decomposition (IOD) of a C*-algebra with respect to an infinite orthogonal family of projections. Let *A* be a unital C*-algebra, *p* be a projection in *A*, i.e. $p^2=p$, $p^*=p$. Then $1-p$ is also a projection and the subsets $pA=\{pa: a\in A\}$, $Ap=\{ap: a\in A\}$, $(1-p)A=\{(1-p)a: a\in A\}$, $A(1-p)=\{a(1-p): a\in A\}$ are vector subspaces of *A*. *A* coincides with its Peirce decomposition on *p*, i.e.

$$ A=pA\oplus Ap\oplus (1-p)A\oplus A(1-p).$$

These subspaces satisfy the following properties

$$\begin{aligned}&pA\cdot pA\subseteq pA, pA\cdot Ap\subseteq pAp,\\ &Ap\cdot Ap\subseteq Ap, pA\cdot (1-p)A\subseteq pA,\\  & (1-p)A\cdot (1-p)A\subseteq (1-p)A, (1-p)A\cdot pA\subseteq (1-p)A,\\  & pA\cdot A(1-p)\subseteq pA(1-p), A(1-p)\cdot pA=\{0\}. \end{aligned}$$

In the present paper an infinite analog of this decomposition, namely, IOD is investigated. In Arzikulov (2008) the notion of IOD is defined as follows: let *A* be a C*-algebra on an infinite dimensional Hilbert space *H*, $\{p_\xi \}$ be an infinite orthogonal family of projections in *A* with the least upper bound (LUB) 1, calculated in *B*(*H*). Let

$$\begin{aligned}&\sum\limits_{\xi ,\eta }^\oplus p_\xi Ap_\eta =\{\{a_{\xi ,\eta }\}: a_{\xi ,\eta }\in p_\xi Ap_\eta \quad for\, all \,\,\xi , \eta , \,and\,there\, exists\, such\, number\\ &\quad K\in R \,\,that\quad  \left\Vert \sum\limits_{k,l=1}^na_{kl}\right\Vert \le K \quad for\, all\,\, n\in N \,\,and\,\,\,\{a_{kl}\}_{kl=1}^n\subseteq \{a_{\xi ,\eta }\}\},\end{aligned}$$

and $\sum\nolimits_{\xi ,\eta }^\oplus p_\xi Ap_\eta$ is said to be an *IOD* of A.

Under this definition the following theorem is valid.

### **Theorem**

(Arzikulov 2008) *Let* *A* be a C*-*algebra on a Hilbert space* *H*, $\{p_\xi \}$ *be an infinite orthogonal family of projections in* *A* *with the least upper bound* 1 *in* *B*(*H*). *Then*

1. *if the order unit space* $\sum\nolimits_{\xi ,\eta }^\oplus p_\xi Ap_\eta$ *is monotone complete in* *B*(*H*)* (i.e.* *ultraweakly closed*), *then* $\sum\nolimits _{\xi ,\eta }^\oplus p_\xi Ap_\eta$ is a C*-algebra,
2. *if* *A* *is monotone complete in* *B*(*H*)* (i.e.* *a von Neumann algebra*), *then* $A=\sum\nolimits _{\xi ,\eta }^\oplus p_\xi Ap_\eta$,
3. *if* $\sum\nolimits _{\xi ,\eta }^\oplus p_\xi Ap_\eta$ is a C*-algebra *then this algebra is a von Neumann algebra*.

In the present paper we give a complete proof of this theorem (see, respectively, item 2 of Theorem 3, Proposition 4, item 2 of Corollary 1).

Also it is proved that an infinite order decomposition (IOD) of a C*-algebra forms the complexification of an order unit space, and, if the C*-algebra is monotone complete (not necessarily weakly closed) then its IOD is also monotone complete ordered vector space. Also it is established that an IOD of a C*-algebra is a C*-algebra if and only if this C*-algebra is a von Neumann algebra. For this propose operations of multiplication and an involution in an IOD are introduced. It turns out, the order and the norm defined in an IOD of a C*-algebra on a Hilbert space *H* coincide with the usual order and norm in *B*(*H*). Also, it is proved that, if a C*-algebra *A* with an infinite orthogonal family $\{p_\xi \}$ of projections in *A* such that $\sup _\xi p_\xi =1$ is not a von Nemann algebra and projections in the set $\{p_\xi \}$ are pairwise equivalent then $A\ne \sum\nolimits _{\xi ,\eta }^\oplus p_\xi Ap_\eta$. Moreover if the Banach space $\sum\nolimits _{\xi ,\eta }^\oplus p_\xi Ap_\eta$ is not weakly closed then $\sum\nolimits _{\xi ,\eta }^\oplus p_\xi Ap_\eta$ is not a C*-algebra. As a result it is proved that an IOD of a C*-algebra forms the complexification of an order unit space. In this sense, if a C*-algebra is monotone complete (and not necessarily weakly closed) then its IOD is monotone complete and an IOD of a C*-algebra is a C*-algebra if and only if this C*-algebra is a von Neumann algebra.

## Infinite order decompositions

A relation of order $\le$ in the vector space $\sum\nolimits _{\xi ,\eta }^\oplus p_\xi Ap_\eta$ we define as follows: for elements $\{a_{\xi \eta }\}$, $\{b_{\xi \eta }\}\in \sum\nolimits _{\xi ,\eta }^\oplus p_\xi Ap_\eta$, if for all $n\in N$, $\{p_k\}_{k=1}^n\subset \{p_\xi \}$ the inequality $\sum\nolimits _{k,l=1}^na_{kl}\le \sum\nolimits _{k,l=1}^nb_{kl}$ is valid, then it will be written $\{a_{\xi \eta }\}\le \{b_{\xi \eta }\}$. Also, the map $\{a_{\xi ,\eta }\}\rightarrow \Vert \{a_{\xi ,\eta }\}\Vert$, $\{a_{\xi ,\eta }\}\in \sum\nolimits _{\xi ,\eta }^\oplus p_\xi Ap_\eta$, where $\Vert \{a_{\xi ,\eta }\}\Vert =\sup \{\Vert \sum\nolimits _{kl=1}^n a_{kl}\Vert :n\in N, \{a_{kl}\}_{kl=1}^n \subseteq \{a_{\xi ,\eta }\}\}$, is a norm on the vector space $\sum\nolimits _{\xi ,\eta }^\oplus p_\xi Ap_\eta$.

### *Example*

Throughout the paper let *n* be an arbitrary infinite cardinal number, $\Xi$ be a set of indices of the cardinality *n*. Let $\{e_{ij}\}$ be the family of matrix units such that $e_{ij}$ is a $n\times n$-dimensional matrix, i.e. $e_{ij}=(a_{\alpha \beta })_{\alpha \beta \in \Xi }$, the (*i*, *j*)-th component of which is 1, i.e. $a_{ij}=1$, and the rest components are zeros. Throughout the paper let

$$\begin{aligned} & M_n({\mathbb{C}})= \,  \left\{\{\lambda ^{ij}e_{ij}\}: \,for\,\, all\,\, indices\,\, i,\,j \,\lambda ^{ij}\in {\mathbb{C}},\right.\\ &{\quad}and\,\, there\,\, exists\,\, such\,\, number\,\, K\in {\mathbf{R}},\,\,that \,\, for \,\, all\,\, n\in N\\ &\left.\quad and\,\, \{e_{kl}\}_{kl=1}^n\subseteq \{e_{ij}\} \left\Vert \sum_{kl=1}^n \lambda ^{kl}e_{kl}\right\Vert \le K\right\}, \end{aligned}$$

where $\Vert \,\, \Vert$ is the norm of a matrix. It is easy to see that $M_n({\mathbb{C}})$ is a vector space. The set $M_n({{\mathbb{C}}})$, defined above, coincides with the set

$$\begin{aligned} &{\mathcal{M}}_n({{\mathbb{C}}})= \left\{\{\lambda _{ij}e_{ij}\}:\,for\,\, all\,\, indexes\, ij\,\,\lambda _{ij}\in {{\mathbb{C}}},\right.\\ &{\quad}and\,\,\, there\,\,\, exists\,\,\, such\,\,\, number\,\,\, K\in R \,\,that \,\, for\,\, all\,\\ &\left.{\quad}\{x_i\}\in l_2(\Xi )\,\, the\,\,\, next\,\,\, inequality\,\,\, is\,valid \, \sum _{j\in \Xi } \left\vert \sum _{i\in \Xi }\lambda _{ij}x_i \right\vert ^2\le K^2\sum _{i\in \Xi } \left\vert x_i\right\vert ^2\right\}, \end{aligned}$$

where $l_2(\Xi )$ is the Hilbert space on ${\mathbb{C}}$ with elements $\{x_i\}_{i\in \Xi }$, where $x_i\in {\mathbb{C}}$ for all $i\in \Xi$.

In the vector space

$${\mathcal{M}}_n({\mathbb{C}})=\{\{\lambda ^{ij}e_{ij}\}: \,for\,\, all\,\, indices\,\, i,\,j \,\lambda ^{ij}\in {\mathbb{C}}\}$$

of all $n\times n$-dimensional *matrices* (indexed sets) over ${\mathbb{C}}$ we can introduce an associative multiplication as follows:

$$ xy=\left\{ \sum _{\xi \in \Xi }\lambda ^{i\xi }\mu ^{\xi j}e_{ij}\right\}, $$

where $x=\{\lambda ^{ij}e_{ij}\}$, $y=\{\mu ^{ij}e_{ij}\}$ are elements of ${\mathcal{M}}_n({\mathbb{C}})$. Then $M_n({\mathbb{C}})$ becomes an associative algebra with respect to this operation and $M_n({\mathbb{C}})\cong B(l_2(\Xi ))$, where $l_2(\Xi )$ is a Hilbert space over ${\mathbb{C}}$ with elements $\{x_i\}_{i\in \Xi }$, $x_i\in {\mathbb{C}}$ for all $i\in \Xi$, $B(l_2(\Xi ))$ is the associative algebra of all bounded linear operators on $l_2(\Xi )$. Hence $M_n({\mathbb{C}})$ is a von Neumann algebra of infinite $n\times n$-dimensional matrices over ${\mathbb{C}}$.

Similarly, if *B*(*H*) is the algebra of all bounded linear operators on a Hilbert space *H* and $\{q_i\}$ is a maximal orthogonal family of minimal projections in *B*(*H*), then $B(H)=\sum\nolimits _{ij}^\oplus q_i B(H)q_j$ (Arzikulov 2008).

Let *A* be a C*-algebra on a Hilbert space *H*, $\{p_i\}$ be an infinite orthogonal family of projections with the LUB 1 in *B*(*H*) and ${\mathcal{A}}=\{ \{p_iap_j\}: a\in A\}$. Then $A\equiv {\mathcal{A}}$ (Arzikulov 2012).

### **Lemma 1**

*Let* *A* be a C*-*algebra on a Hilbert space* *H*, $\{p_\xi \}$ *be an infinite orthogonal family of projections in* *A* *with the LUB* 1 *in* *B*(*H*). *Then* $\sum\nolimits _{\xi ,\eta }^\oplus p_\xi Ap_\eta$ *is a vector space with the following componentwise* *algebraic operations*

$$\begin{aligned} &\lambda \cdot \{a_{\xi \eta }\}=\{ \lambda a_{\xi \eta }\},\quad \lambda \in {\mathbb{C}}\\ &\{a_{\xi \eta }\}+\{b_{\xi \eta }\}=\{a_{\xi \eta }+b_{\xi \eta }\},\quad a_{\xi \eta }, b_{\xi \eta }\in \sum _{\xi ,\eta }^\oplus p_\xi Ap_\eta \end{aligned}$$

*and* ${\mathcal{A}}$ *is a vector subspace of* $\sum\nolimits _{\xi ,\eta }^\oplus p_\xi Ap_\eta$.

### **Lemma 2**

*Let* *A* be a C*-*algebra on a Hilbert space* *H*, $\{p_\xi \}$ *be an* *infinite orthogonal family of projections in* *A* *with the LUB* 1 *in* *B*(*H*). *Then the map* $\{a_{\xi ,\eta }\}\rightarrow \Vert \{a_{\xi ,\eta }\}\Vert$, $\{a_{\xi ,\eta }\}\in \sum\nolimits _{\xi ,\eta }^\oplus p_\xi Ap_\eta$, *where* $\Vert \{a_{\xi ,\eta }\}\Vert =\sup \{\Vert \sum\nolimits _{kl=1}^n a_{kl}\Vert :n\in N, \{a_{kl}\}_{kl=1}^n \subseteq \{a_{\xi ,\eta }\}\}$, *is a norm and* $\sum\nolimits _{\xi ,\eta }^\oplus p_\xi Ap_\eta$ *is a Banach space with this norm*.

### *Proof*

It is clear, that for every element $\{a_{\xi ,\eta }\}\in \sum\nolimits _{\xi ,\eta }^\oplus p_\xi Ap_\eta$, if $\Vert \{a_{\xi ,\eta }\}\Vert =0$, then $a_{\xi ,\eta }=0$ for all $\xi$, $\eta$, i.e. $\{a_{\xi ,\eta }\}$ is the zero element. The other conditions in the definition of the norm can be also easily checked.

Let $(a_n)$ be a Cauchy sequence in $\sum\nolimits _{\xi ,\eta }^\oplus p_\xi Ap_\eta$, i.e. for each positive number $\varepsilon >0$ there exists $n\in {\mathbb{N}}$ such, that $\Vert a_{n_1}-a_{n_2}\Vert <\varepsilon$ for all $n_1\ge n$, $n_2\ge n$. Then the set $\{\Vert a_n\Vert \}$ is bounded by some number $K\in {\mathbb{R}}_+$ and for every finite set $\{p_k\}_{k=1}^n\subset \{p_i\}$ the sequence $(pa_np)$ is a Cauchy sequence, where $p=\sum\nolimits _{k=1}^n p_k$. Then, $\lim _{n\rightarrow \infty } pa_np\in A$ since *A* is a Banach space.

Let $a_{\xi ,\eta }=\lim _{n\rightarrow \infty } p_\xi a_np_\eta$ for all $\xi$ and $\eta$. Then $\Vert \sum\nolimits _{kl=1}^n a_{kl}\Vert \le K$ for all $n\in {\mathbb{N}}$ and $\{a_{kl}\}_{kl=1}^n \subseteq \{a_{\xi ,\eta }\}$. Hence $\{a_{\xi ,\eta }\}\in \sum\nolimits _{\xi ,\eta }^\oplus p_\xi Ap_\eta$. $\square$

The definition of the order in $\sum\nolimits _{\xi ,\eta }^\oplus p_\xi Ap_\eta$ is equivalent to the following condition: for the elements $\{a_{\xi \eta }\}$, $\{b_{\xi \eta }\}\in \sum\nolimits _{\xi ,\eta }^\oplus p_\xi Ap_\eta$, if $\{a_{kl}\}_{k,l=1}^n\le \{b_{kl}\}_{k,l=1}^n$ for all $n\in N$ and $\{p_k\}_{k=1}^n \subseteq \{p_i\}$ in ${\mathcal{A}}$, then $\{a_{\xi \eta }\}\le \{b_{\xi \eta }\}$. Let $\{a_{\xi \eta }\}^*=\{a_{\eta \xi }^*\}$ for every $\{a_{\xi \eta }\}\in \sum\nolimits _{\xi \eta }^\oplus p_\xi Ap_\eta$ and $(\sum\nolimits _{\xi \eta }^\oplus p_\xi Ap_\eta )_{sa}=\{\{a_{\xi \eta }\}: \{a_{\xi \eta }\}\in \sum\nolimits _{\xi \eta }^\oplus p_\xi Ap_\eta , \{a_{\xi \eta }\}^*=\{a_{\xi \eta }\}\}$.

### **Proposition 1**

*Let* *A* be a C*-*algebra on a Hilbert space* *H*, $\{p_\xi \}$ *be an infinite orthogonal family of projections in* *A* *with the LUB* 1 *in* *B*(*H*). *Then the relation* $\le$, *introduced above, is a* *relation of partial order, and* $(\sum\nolimits _{\xi ,\eta }^\oplus p_\xi Ap_\eta )_{sa}$ *is an order unit space with this order. In this case* ${\mathcal{A}}_{sa}=\{\{p_\xi ap_\eta \}: a\in A_{sa}\}$ is an order *unit subspace of* $(\sum\nolimits _{\xi ,\eta }^\oplus p_\xi Ap_\eta )_{sa}$.

### *Proof*

Let ${\mathcal {M}}=(\sum\nolimits _{\xi ,\eta }^\oplus p_\xi Ap_\eta )_{sa}$. ${\mathcal{M}}$ is a partially ordered vector space, i.e. ${\mathcal{M}}_+\cap {\mathcal{M}}_-=\{0\}$, where ${\mathcal{M}}_+=\{ \{a_{\xi \eta }\}\in {\mathcal{M}}: \{a_{\xi \eta }\}\ge 0\}$, ${\mathcal{M}}_-=\{ \{a_{\xi \eta }\}\in {\mathcal{M}}: \{a_{\xi \eta }\}\le 0\}$.

By the definition of the order ${\mathcal{M}}$ is Archimedean. Let $\{a_{\xi \eta }\}\in {\mathcal{M}}$. Since $-\Vert \{a_{\xi ,\eta }\}\Vert p\le p\{a_{\xi ,\eta }\}p\le \Vert \{a_{\xi ,\eta }\}\Vert p$ for every finite set $\{p_k\}_{k=1}^n\subset \{p_\xi \}$, where $p=\sum\nolimits _{k=1}^n p_k$, we have $-\Vert \{a_{\xi ,\eta }\}\Vert 1\le \{a_{\xi ,\eta }\}\le \Vert \{a_{\xi ,\eta }\}\Vert 1$ by the definition of the order, and the unit of *A* is an order unit of the partially ordered vector space ${\mathcal{M}}$. Thus ${\mathcal{M}}$ is an order unit space.

By Lemma 1 ${\mathcal{A}}$ is an order unit subspace of the order unit space ${\mathcal{M}}$. $\square$

### **Proposition 2**

Let A be a C*-algebra on a Hilbert space H, $\{p_i\}$ *be an infinite orthogonal family of projections in A with the LUB 1 in B(H). Then* ${\mathcal{A}}=\{\{p_\xi ap_\eta \}: a\in A\}$ is a C*-algebra, where the operation of multiplication of ${\mathcal{A}}$ *is defined as follows*

$$\cdot :\langle\{p_\xi ap_\eta \},\{p_\xi bp_\eta \}\rangle\rightarrow \{p_\xi abp_\eta \}, \{p_\xi ap_\eta \},\{p_\xi bp_\eta \}\in {\mathcal{A}}. $$

### *Proof*

By Lemma 4 in Arzikulov (2012) the map

$${\mathcal{I}}: a\in A\rightarrow \{p_\xi ap_\eta \}\in {\mathcal{A}} $$

is a one-to-one map. In this case

$${\mathcal {I}}(a){\mathcal{I}}(b)={\mathcal{I}}(ab)$$

by the definition of the operation of multiplication in Proposition 2, and ${\mathcal{I}}(a)=\{p_\xi ap_\eta \}$, ${\mathcal{I}}(b)=\{p_\xi bp_\eta \}$, ${\mathcal{I}}(ab)=\{p_\xi ab p_\eta \}$. Hence, the operation, introduced in Proposition 2 is associative multiplication and the map ${\mathcal{I}}$ is an isomorphism of the algebras *A* and ${\mathcal{A}}$.

By Proposition 1 the isomorphism ${\mathcal{I}}$ is isometrical. Therefore ${\mathcal{A}}$ is a C*-algebra with this operation. $\square$

### *Example 1*

Let *H* be a Hilbert space, $\{q_i\}$ be a maximal orthogonal family of minimal projections in *B*(*H*). Then $\sup _i q_i=1$ and by Lemma 4 in Arzikulov (2012) and Proposition 2 the algebra ${\mathcal{B(H)}}=\{\{q_iaq_j\}: a\in B(H)\}$ can be identified with *B*(*H*) as a C*-algebra in the sense of the map

$${\mathcal {I}}: a\in B(H)\rightarrow \{q_iaq_j\}\in {\mathcal{B(H)}}. $$

In this case associative multiplication in ${\mathcal{B(H)}}$ is defined as follows

$$\cdot :\langle\{q_i aq_j\},\{q_i bq_j\}\rangle\rightarrow \{q_i abq_j\}, \{q_i aq_j\},\{q_i bq_j\}\in {\mathcal{B(H)}}.$$

Let *a*, $b\in B(H)$, $q_iaq_j=\lambda _{ij}q_{ij}$, $q_ibq_j=\mu _{ij}q_{ij}$, where $\lambda _{ij}$, $\mu _{ij}\in {\mathbf{C}}$, $q_i=q_{ij}q_{ij}^*$, $q_j=q_{ij}^*q_{ij}$, for all indices *i* and *j*. Then this operation of multiplication coincides with the following bilinear operation

$$\cdot :\langle\{q_i aq_j\},\{q_i bq_j\}\rangle\rightarrow \left\{\sum _\xi \lambda _{i\xi } \mu _{\xi j}q_{ij}\right\}, \{q_i aq_j\},\{q_i bq_j\}\in {\mathcal{B(H)}}.$$

### *Remark 1*

Let *A* be a C*-algebra on a Hilbert space *H*, $\{p_i\}$ be an infinite orthogonal family of projections in *A* with the LUB 1 in *B*(*H*). Then by Proposition 2 ${\mathcal{A}}=\{\{p_\xi ap_\eta \}: a\in A\}$ is a C*-algebra. In this case the operation of involution on the algebra ${\mathcal{A}}$ coincides with the map

$$ \{p_\xi ap_\eta \}^*=\{p_\xi a^*p_\eta \},\quad a\in A. $$

Indeed, the identification ${\mathcal{A}}\equiv A$ gives $a=\{p_\xi ap_\eta \}$ and $a^*=\{p_\xi a^*p_\eta \}$ for all $a\in A$. Then $\{p_\xi ap_\eta \}^*=a^*=\{p_\xi a^*p_\eta \}$ for each $a\in A$. Let ${\mathcal{A}}_{sa}=\{\{p_\xi ap_\eta \}: a\in A_{sa}\}$. Then ${\mathcal{A}}={\mathcal{A}}_{sa}+i {\mathcal{A}}_{sa}$. Indeed, $\{p_\xi ap_\eta \}^*=a^*=a=\{p_\xi ap_\eta \}$ for each $a\in A_{sa}$.

Let ${\mathcal{N}}=\{\{p_\xi ap_\eta \}: a\in B(H)\}$. By Lemma 4 in Arzikulov (2012) and by Proposition 2 ${\mathcal{N}}\equiv B(H)$. Therefore it will be assumed that ${\mathcal{N}}=B(H)$. Let ${\mathcal{N}}_{sa}=\{\{p_\xi ap_\eta \}: a\in B(H), \{p_\xi ap_\eta \}^*=\{p_\xi ap_\eta \}\}$. Then ${\mathcal{N}}={\mathcal{N}}_{sa}+i{\mathcal{N}}_{sa}$. Note that $\{p_\xi ap_\eta \}^*=\{p_\xi ap_\eta \}$ if and only if $(p_\xi ap_\eta )^*=p_\eta ap_\xi$ for all $\xi$, $\eta$.

### **Lemma 3**

*Let* *H* *be a Hilbert space*, $\{p_\xi \}$ *be an infinite orthogonal* *family of projections in* *B*(*H*) *with the LUB 1. Then associative multiplication of the algebra* ${\mathcal{N}}$ (*hence of the algebra* *B*(*H*)) *coincides with the operation*

$$\{p_\xi ap_\eta \}\star \{p_\xi bp_\eta \}=\left\{\sum _i p_\xi ap_i p_i bp_\eta \right\}, \{p_\xi ap_\eta \},\{p_\xi bp_\eta \}\in {\mathcal {N}} $$

*where the sum* $\sum$ *in the right part of the equality is an ultraweak limit of the net of finite sums of elements in the set* $\{p_\xi ap_i p_i bp_\eta \}_{\xi \eta }$.

### *Proof*

Let $\{p_k\}_{k=1}^n$ be a finite subset of the set $\{p_\xi \}$. Note that $\sup _i p_i=1$, i.e. the net of all finite sums $\sum\nolimits _{k=1}^n p_k$ of orthogonal projections in $\{p_\xi \}$ ultraweakly converges to the identity operator in *B*(*H*). By the ultraweakly continuity of the operator of multiplication $T(b)=ab, b\in B(H)$, where $a\in B(H)$, the net of finite sums of elements in $\{p_\xi ap_i p_i bp_\eta \}_{\xi \eta }$ ultraweakly converges and $\sum\nolimits _i p_\xi ap_i p_i bp_\eta =p_\xi abp_\eta$ for all $\xi$, $\eta$. Hence the operation of multiplication $\star$ of the algebra ${\mathcal{N}}$ coincides with the operation, introduced in Proposition 2. And the operation of associative multiplication, introduced in Proposition 2 coincides with multiplication in *B*(*H*) in the sense ${\mathcal{N}}\equiv B(H)$. $\square$

### **Proposition 3**

*Let* *A* be a C*-*algebra on a Hilbert space* *H*, $\{p_\xi \}$ *be an infinite orthogonal family of projections in* *A* *with the LUB 1 in B(H). Then the operation of associative multiplication of* ${\mathcal{A}}$ *coincides with the operation of associative multiplication of* ${\mathcal{N}}$ *on* *A*, *defined in Lemma* 3.

### *Proof*

Let $\{p_\xi ap_\eta \}$, $\{p_\xi bp_\eta \}$ be elements of ${\mathcal{A}}_{sa}$ and $\{p_k\}_{k=1}^n$ be a finite subset of the set $\{p_\xi \}$ and $p=\sum\nolimits _{k=1}^n p_k$. The net of all finite sums $\sum\nolimits _{k=1}^n p_k$ of orthogonal projections in $\{p_\xi \}$ ultraweakly converges to the identity operator in *B*(*H*). Therefore for all $\xi$, $\eta$ the element $\{p_\xi abp_\eta \}$ is an ultraweak limit in *B*(*H*) of the net $\{\sum\nolimits _i p_\xi ap_i p_i bp_\eta \}$ of all finite sums $\{\sum\nolimits _{k=1}^n p_\xi ap_k p_k bp_\eta \}$ for all finite subsets $\{p_k\}_{k=1}^n\subset \{p_\xi \}$, and the element $\{p_\xi abp_\eta \}$ belongs to ${\mathcal{A}}$. Hence the assertion of Proposition 3 is valid. $\square$

### *Remark 2*

Let *A* be a C*-algebra on a Hilbert space *H*, $\{p_i\}$ be an infinite orthogonal family of projections in *A* with the LUB 1 in *B*(*H*). Then by Lemmata 3, 4 in Arzikulov (2012) the order and the norm in the vector space $\sum\nolimits _{i,j}^\oplus p_i Ap_j$ can be introduced as follows: $\{a_{ij}\}\ge 0$ denotes that this element is zero or positive element in *B*(*H*) in the sense $B(H)=\sum\nolimits _{\xi ,\eta }^\oplus q_\xi B(H)q_\eta$, where $\{q_\xi \}$ is an arbitrary maximal orthogonal family of minimal projections in *B*(*H*); $\Vert \{a_{ij}\}\Vert$ is equal to the norm in *B*(*H*) of this element in the sense of the equality $B(H)=\sum\nolimits _{\xi ,\eta }^\oplus q_\xi B(H)q_\eta$ (Example 1). By Lemmata 3 and 4 in Arzikulov (2012) they coincide with the order and the norm defined in Lemma 2 and Proposition 1, respectively. If *a* is a bounded linear operator on *H* then

$$ a=\sum _{\xi ,\eta }^\oplus q_\xi a q_\eta,$$

where $\sum\nolimits _{\xi ,\eta }^\oplus q_\xi a q_\eta$ is the ultraweak limit of the net of finite sums. By Lemma 2, if $A=B(H)$, then

$$\Vert a\Vert =\sup \left\{\left\Vert \sum _{kl=1}^n q_k a q_l\right\Vert :n\in N, \{q_k a q_l\right\}_{kl=1}^n \subseteq \{q_\xi a q_\eta \}\}.$$

If $H=l_2(\Xi )$, where $l_2(\Xi )$ is the Hilbert space on ${\mathbb{C}}$ with elements $\{x_i\}_{i\in \Xi }$, $x_i\in {\mathbf{C}}$ for all $i\in \Xi$, then $B(H)=B(l_2(\Xi ))$, where $B(l_2(\Xi ))$ is the associative algebra of all bounded linear operators on the Hilbert space $l_2(\Xi )$, which is an associative algebra of infinite dimensional matrices. In this case $\Vert a\Vert$ is a supremum of norms of all finite-dimensional main diagonal submatrices of *a*. Hence the following theorem is valid.

### **Theorem 1**

*The norm of an infinite dimensional matrix is equal to the supremum of norms of all finite dimensional main diagonal submatrices of this matrix.*

By Lemma 3 in Arzikulov (2012) the following theorem is also valid.

### **Theorem 2**

*An infinite dimensional matrix is positive if and only if all finite dimensional main diagonal submatrices of this matrix are positive.*

It should be noted that Theorem 1 of $\S$ 50 in Berberian (1972) follows from Theorem 2.

### *Remark 3*

Suppose that all conditions of Remark 3 are valid. Let ${\mathcal{B(H)}}=\sum\nolimits _{\xi ,\eta }^\oplus q_\xi B(H)q_\eta$. Then $B(H)\equiv {\mathcal {B(H)}}$, where ${\mathcal{B(H)}}=\{\{q_\xi aq_\eta \}: a\in B(H)\}$. Also, $\sum\nolimits _{ij}^\oplus p_i Ap_j$ is a Banach space and an order unit space (Lemma 2, Proposition 1). Suppose that $\{q_\xi \}$ is a maximal orthogonal family of minimal projections in *B*(*H*) such that $p_i=\sup _\eta q_\eta$ for some subset $\{q_\eta \}\subset \{q_\xi \}$ for all *i*. Note that $B(H)\equiv \{\{p_iap_j\}: a\in B(H)\}=\sum\nolimits _{ij}^\oplus p_i B(H)p_j$. By Propositions 2 and 3 the order unit space ${\mathcal{A}}=\{\{p_iap_j\}: a\in A\}$ is closed with respect to the associative multiplication of $\sum\nolimits _{ij}^\oplus p_i B(H)p_j$ (i.e. ${\mathcal{N}}=\{\{p_iap_j\}: a\in B(H)\}$).

At the same time, the order unit space $\sum\nolimits _{ij}^\oplus p_i Ap_j$ is the order unit subspace of $\sum\nolimits _{ij}^\oplus p_i B(H)p_j$.

Since $B(H)\equiv \sum\nolimits _{ij}^\oplus p_i B(H)p_j$ we have $\sum\nolimits _{ij}^\oplus p_i B(H)p_j$ is a von Neumann algebra, and, without loss of generality, this algebra can be considered as *B*(*H*).

Note that if $\sum\nolimits _{ij}^\oplus p_i Ap_j$ is closed with respect to the associative multiplication of $\sum\nolimits _{ij}^\oplus p_i B(H)p_j$, then $\sum\nolimits _{ij}^\oplus p_i Ap_j$ is a C*-algebra. Also, if *A* is the C*-algebra with the conditions, which are listed above, then the vector space $\sum\nolimits _{ij}^\oplus p_i Ap_j$ is an order unit subspace of $\sum\nolimits _{ij}^\oplus p_i B(H)p_j$. Then

$${\mathcal{A}}\subseteq \sum _{ij}^\oplus p_i Ap_j\subseteq \sum _{ij}^\oplus p_i B(H)p_j.$$

Thus, further the statement that $\sum\nolimits _{ij}^\oplus p_i Ap_j$ is a C*-algebra denotes $\sum\nolimits _{ij}^\oplus p_i Ap_j$ is closed with respect to the associative multiplication of $\sum\nolimits _{ij}^\oplus p_i B(H)p_j$.

The involution in $\sum\nolimits _{ij}^\oplus p_i B(H)p_j$ in the sense of the identification $\sum\nolimits _{ij}^\oplus p_i B(H)p_j\equiv B(H)$ coincides with the map

$$ \{a_{ij}\}^*=\{a_{ji}^*\}, \{a_{ij}\}\in \sum _{ij}^\oplus p_i B(H)p_j.$$

Indeed, there exists an element $a\in B(H)$ such that $a=\{a_{ij}\}=\{p_iap_j\}$. Then $a^*=\{p_ia^*p_j\}$ in the sense of $B(H)\equiv {\mathcal{N}}$ and $a_{ij}=p_iap_j$, $a_{ij}^*=p_ja^*p_i$ for all *i*, *j*. Therefore $\{p_ia^*p_j\}=\{a_{ji}^*\}$. Hence $a^*=\{a_{ji}^*\}$. Let $(\sum\nolimits _{ij}^\oplus p_i B(H)p_j)_{sa}=\{\{a_{ij}\}: \{a_{ij}\}\in \sum\nolimits _{ij}^\oplus p_i B(H)p_j, \{a_{ij}\}^*=\{a_{ij}\}\}$. Then

$$ \sum _{ij}^\oplus p_i B(H)p_j=\left(\sum _{ij}^\oplus p_i B(H)p_j)_{sa}+i(\sum _{ij}^\oplus p_i B(H)p_j\right)_{sa}. $$

### **Lemma 4**

*Let* *A* be a C*-*algebra on a Hilbert space* *H*, $\{p_i\}$ *be an infinite orthogonal family of projections in* *A* *with LUB* 1 *in* *B*(*H*) *and* $(\sum\nolimits _{ij}^\oplus p_i Ap_j)_{sa}=\{\{a_{ij}\}: \{a_{ij}\}\in \sum\nolimits _{ij}^\oplus p_i Ap_j, \{a_{ij}\}^*=\{a_{ij}\}\}$. *Then*

1 $$ \sum _{ij}^\oplus p_i Ap_j=\left(\sum _{ij}^\oplus p_i Ap_j\right)_{sa}+i\left(\sum _{ij}^\oplus p_i Ap_j\right)_{sa}.$$

*In this case* $\{a_{ij}\}^*=\{a_{ij}\}$ *if and only if* $a_{ij}^*=a_{ji}$ *for all* *i*, *j*.

### *Proof*

Let $\{a_{ij}\}\in \sum\nolimits _{ij}^\oplus p_i Ap_j$. Since $a_{ij}+a_{ji}\in A$, we have $a_{ij}+a_{ji}=a_1+ia_2$, where $a_1$, $a_2\in (\sum\nolimits _{ij}^\oplus p_i Ap_j)_{sa}$, for all *i* and *j*. Then $a_{ij}+a_{ji}=p_ia_1p_j+p_ja_1p_i+i(p_ia_2p_j+p_ja_2p_i)$, $a_1=p_ia_1p_j+p_ja_1p_i$, $a_2=p_ia_2p_j+p_ja_2p_i$ for all *i* and *j*. Let $a^1_{ij}=p_ia_1p_j+p_ja_1p_i$, $a^2_{ij}=p_ia_2p_j+p_ja_2p_i$ for all *i* and *j*. Then $\{a^1_{ij}\}$, $\{a^2_{ij}\}\in \sum\nolimits _{ij}^\oplus p_i Ap_j$ by the definition of $\sum\nolimits _{ij}^\oplus p_i Ap_j$. In this case $\{a^k_{ij}\}^*=\{a^k_{ij}\}$, $k=1,2$. Since $\{a_{ij}\}\in \sum\nolimits _{ij}^\oplus p_i Ap_j$ was chosen arbitrarily, we have the equality (1).

The rest part of Lemma 4 is valid by the definition of the self-adjoint elements $\{a^k_{ij}\}$, $k=1,2$. $\square$

### **Lemma 5**

*Let* *H* *be a Hilbert space*, $\{p_\xi \}$ *be an infinite orthogonal family of projections in* *B*(*H*) *with the LUB* 1. *Then the operation of associative multiplication of the* *algebra* $\sum\nolimits _{\xi ,\eta }^\oplus p_\xi B(H)p_\eta$ (i.e. *of the algebra* *B*(*H*)) *coincides with the binary operation*

2 $$ \cdot :\langle\{a_{\xi ,\eta }\},\{b_{\xi ,\eta }\}\rangle\rightarrow \left\{\sum _i a_{\xi i} b_{i\eta }\}, \{a_{\xi \eta } \right\},\{b_{\xi \eta }\}\in \left(\sum _{\xi ,\eta }^\oplus p_\xi B(H)p_\eta\right).$$

### *Proof*

Let $$\{a_{\xi \eta }\},\{b_{\xi \eta } \}\in (\sum\nolimits _{\xi ,\eta }^\oplus p_\xi B(H)p_\eta )$$. By

$$B(H)\equiv {\mathcal{N}}\equiv \sum _{\xi ,\eta }^\oplus p_\xi B(H)p_\eta.$$

it can be admitted that $B(H)={\mathcal{N}}=\sum\nolimits _{\xi ,\eta }^\oplus p_\xi B(H)p_\eta$. There exists elements *a*, *b* in *B*(*H*) such that $p_\xi ap_\eta =a_{\xi \eta }$, $p_\xi bp_\eta =b_{\xi \eta }$ for all $\xi$, $\eta$. Therefore $\{a_{\xi \eta }\}=\{p_\xi ap_\eta \}$, $\{b_{\xi \eta }\}=\{p_\xi bp_\eta \}$. Then by Lemma 3 the associative multiplication of $\sum\nolimits _{\xi ,\eta }^\oplus p_\xi B(H)p_\eta$ (i.e. of *B*(*H*)) coincides with binary operation (2). $\square$

### **Proposition 4**

(Arzikulov 2008) *Let* *A* *be a von Neumann algebra on a Hilbert space* *H*, $\{p_i\}$ *be an infinite orthogonal family of projections in* *A* *with LUB* 1. *Then* $A=\sum\nolimits _{\xi ,\eta }^\oplus p_\xi Ap_\eta$.

### *Proof*

Let *a* be an element of $\sum\nolimits _{\xi ,\eta }^\oplus p_\xi Ap_\eta$ and $a=\{a_{\xi \eta }\}$, where $a_{\xi \xi }=p_\xi ap_\xi$, $a_{\xi \eta }=p_\xi ap_\eta$ for all $\xi$, $\eta$. Then $a\in B(H)=\sum\nolimits _{\xi ,\eta }^\oplus p_\xi B(H)p_\eta$ and $(\sum\nolimits _{k=1}^n p_k)a(\sum\nolimits _{k=1}^n p_k)\in A$ for every $\{p_k\}_{k=1}^n\subset \{p_\xi \}$. Let

$$ b_n^\alpha =\sum _{kl=1}^n p_k^\alpha ap_l^\alpha =\left(\sum _{kl=1}^n p_k^\alpha\right)a\left(\sum _{kl=1}^n p_k^\alpha\right)$$

for all natural numbers *n* and finite subsets $\{p_k^\alpha \}_{k=1}^n\subset \{p_i\}$. Then by the proof of Lemma 3 in Arzikulov (2012) the net $(b_n^\alpha )$ ultraweakly converges to *a* in *B*(*H*). At the same time *A* is ultraweakly closed in *B*(*H*). Therefore $a\in A$ and $\sum\nolimits _{\xi ,\eta }^\oplus p_\xi Ap_\eta \subseteq A$. $\square$

### **Lemma 6**

*Let* *A* be a C*-*algebra on a Hilbert space* *H*, $\{p_\xi \}$ *be an infinite orthogonal family of projections in* *A* *with the LUB* 1 *in* *B*(*H*). *Then, if projections in* $\{p_\xi \}$ *are pairwise equivalent and* $p_\xi Ap_\xi$ *is a von Neumann algebra for every index* $\xi$, *then* $\sum\nolimits _{\xi ,\eta }^\oplus p_\xi Ap_\eta$ *is closed with respect to the multiplication of the algebra* $\sum\nolimits _{\xi ,\eta }^\oplus p_\xi B(H)p_\eta$ *and* $\sum\nolimits _{\xi ,\eta }^\oplus p_\xi Ap_\eta$ is a C*-*algebra*.

### *Proof*

First, note that $(p_\xi +p_\eta )A(p_\xi +p_\eta )$ is a von Neumann algebra. Indeed, for each net $(a_\alpha )$ in $p_\xi Ap_\eta$, weakly converging in *B*(*H*) the net $(a_\alpha x_{\xi \eta }^*)$ belongs to $p_\xi Ap_\xi$, where $x_{\xi \eta }$ is an isometry in *A* such that $x_{\xi \eta }x_{\xi \eta }^*=p_\xi$, $x_{\xi \eta }^*x_{\xi \eta }=p_\eta$. Then, since the net $(a_\alpha x_{\xi \eta }^*)$ weakly converges in *B*(*H*), we have the weak limit *b* in *B*(*H*) of the net $(a_\alpha x_{\xi \eta }^*)$ belongs to $p_\xi Ap_\xi$. Hence $bx_{\xi \eta }\in p_\xi Ap_\eta$. It is easy to see that $bx_{\xi \eta }$ is a weak limit in *B*(*H*) of the net $(a_\alpha )$. Hence $p_\xi Ap_\eta$ is weakly closed in *B*(*H*).

Let $$\{a_{\xi \eta }\}$$, $$\{b_{\xi \eta }\}\in (\sum\nolimits _{\xi \eta }^\oplus p_\xi Ap_\eta )$$. By

$$ \sum _{\xi \eta }^\oplus p_\xi Ap_\eta \subseteq \sum _{\xi \eta }^\oplus p_\xi B(H)p_\eta =B(H)$$

there exist elements *a*, *b* in $\sum\nolimits _{\xi \eta }^\oplus p_\xi B(H)p_\eta$ (i.e. in *B*(*H*)) such that $p_\xi ap_\eta =a_{\xi \eta }$, $p_\xi bp_\eta =b_{\xi \eta }$ for all $\xi$, $\eta$. Therefore $\{a_{\xi \eta }\}=\{p_\xi ap_\eta \}$, $\{b_{\xi \eta }\}=\{p_\xi bp_\eta \}$. Hence

$$ \sum _i a_{\xi i} b_{i\eta }=p_\xi abp_\eta , $$

calculated in $\sum\nolimits _{\xi \eta }^\oplus p_\xi B(H)p_\eta$, belongs to $p_\xi Ap_\eta$. Since the indices $\xi$, $\eta$ were chosen arbitrarily and the product $\{p_\xi ap_\eta \}\{p_\xi bp_\eta \}=ab$ belongs to $\sum\nolimits _{\xi \eta }^\oplus p_\xi B(H)p_\eta$, we have the product of the elements *a* and *b* belongs to $\sum\nolimits _{\xi ,\eta }^\oplus p_\xi Ap_\eta$. Therefore $\sum\nolimits _{\xi \eta }^\oplus p_\xi Ap_\eta$ is closed with respect to the associative multiplication of $\sum\nolimits _{\xi \eta }^\oplus p_\xi B(H)p_\eta$. At the same time, $\sum\nolimits _{\xi \eta }^\oplus p_\xi Ap_\eta$ is a norm closed subspace of $\sum\nolimits _{\xi \eta }^\oplus p_\xi B(H)p_\eta =B(H)$. Hence $\sum\nolimits _{\xi \eta }^\oplus p_\xi Ap_\eta$ is a C*-algebra and the operation of multiplication in $\sum\nolimits _{\xi \eta }^\oplus p_\xi Ap_\eta$ can be defined as in Lemma 5. $\square$

### **Theorem 3**

*Let* A be a C*-algebra on a Hilbert space *H*, $\{p_\xi \}$ *be an infinite orthogonal family of projections in A with the LUB 1 in* *B* *(H). Then the following statements are valid:*

1. *Suppose that projections in* $\{p_\xi \}$ *are pairwise equivalent and for each* $\xi$ $p_\xi Ap_\xi$ *is a von Nemann algebra. Then* $\sum\nolimits _{\xi ,\eta }^\oplus p_\xi Ap_\eta$ *is a von Neumann algebra,*
2. *if* $\sum\nolimits _{\xi ,\eta }^\oplus p_\xi Ap_\eta$ *is monotone complete in B(H) then* $\sum\nolimits _{\xi ,\eta }^\oplus p_\xi Ap_\eta$ is a C*-algebra.

### *Proof*

(1) Let $\{x_{\xi \eta }\}$ be a set of isometries in *A* such that $p_\xi =x_{\xi \eta }x_{\xi \eta }^*$, $p_\eta =x_{\xi \eta }^*x_{\xi \eta }$ for all $\xi$, $\eta$. Let $\xi$, $\eta$ be arbitrary indices. We prove that $p_\xi Ap_\eta$ is weakly closed. Let $(a_\alpha )$ be a net in $p_\xi Ap_\eta$, weakly converging to an element *a* in *B*(*H*). Then the exists a net $(b_\alpha )$ in $p_\xi Ap_\eta$ such that $a_\alpha =x_{\xi \eta }b_\alpha x_{\xi \eta }$ for all $\alpha$. By separately weakly continuity of the multiplication the net $(a_\alpha x_{\xi \eta }^*)$ weakly converges to $ax_{\xi \eta }$ in *B*(*H*). Since $(a_\alpha x_{\xi \eta }*)\subset p_\xi Ap_\xi$ and $p_\xi Ap_\xi$ is weakly closed in *B*(*H*) we have $ax_{\xi \eta }^*\in p_\xi Ap_\xi$. Hence there exists an element $b\in A$ such that $ax_{\xi \eta }^*=x_{\xi \eta }bx_{\xi \eta }x_{\xi \eta }^*$. Then $ax_{\xi \eta }^*x_{\xi \eta }=x_{\xi \eta }bx_{\xi \eta }x_{\xi \eta }^*x_{\xi \eta } =x_{\xi \eta }bx_{\xi \eta }p_\eta =x_{\xi \eta }bx_{\xi \eta }\in p_\xi Ap_\eta$. At the same time $a_\alpha p_\eta =a_\alpha$ for all $\alpha$. Hence, $ap_\eta =a$. Since $a=ax_{\xi \eta }^*x_{\xi \eta }=x_{\xi \eta }bx_{\xi \eta }\in p_\xi Ap_\eta$ we have $a\in p_\xi Ap_\eta$. Also, since the net $(a_\alpha )$ is chosen arbitrarily we obtain the component $p_\xi Ap_\eta$ is weakly closed in *B*(*H*). Let $(a_\alpha )$ be a net in $\sum\nolimits _{\xi ,\eta }^\oplus p_\xi Ap_\eta$, weakly converging to an element *a* in *B*(*H*). Then for all $\xi$ and $\eta$ the net $(p_\xi a_\alpha p_\eta )$ weakly converges to $p_\xi ap_\eta$ in *B*(*H*). In this case, by the previous part of the proof $p_\xi ap_\eta \in p_\xi Ap_\eta$ for all $\xi$, $\eta$. Note that $a\in \sum\nolimits _{\xi ,\eta }^\oplus p_\xi B(H)p_\eta$. Hence $a\in \sum\nolimits _{\xi ,\eta }^\oplus p_\xi Ap_\eta$. Since the net $(a_\alpha )$ is chosen arbitrarily we have $\sum\nolimits _{\xi ,\eta }^\oplus p_\xi Ap_\eta$ is weakly closed in $\sum\nolimits _{\xi ,\eta }^\oplus p_\xi B(H)p_\eta \equiv B(H)$. Therefore by Lemma 6 $\sum\nolimits _{\xi ,\eta }^\oplus p_\xi Ap_\eta$ is a von Neumann algebra.

Item (2) follows from (1). $\square$

### **Proposition 5**

Let A be a monotone complete C*-algebra on a Hilbert space H, $\{p_\xi \}$ *be an infinite orthogonal family of projections in A with the LUB 1 in B(H). Then the order unit space* $\sum\nolimits _{\xi ,\eta }^\oplus p_\xi Ap_\eta$ *is monotone complete.*

### *Proof*

It is clear that the C*-subalgebra $p_\xi Ap_\xi$ is also monotone complete for each $\xi$. Let $\{p_k\}_{k=1}^n$ be a finite subset of $\{p_\xi \}$ and $p=\sum\nolimits _{k=1}^n p_k$. Then the C*-subalgebra *pAp* is also monotone complete.

Let $(a_\alpha )$ be a bounded monotone increasing net in $\sum\nolimits _{\xi ,\eta }^\oplus p_\xi Ap_\eta$. Since for every $\{p_k\}_{k=1}^n\subseteq \{p_\xi \}$ the subalgebra $(\sum\nolimits _{k=1}^n p_k) A(\sum\nolimits _{k=1}^n p_k)$ is monotone complete we have

$$ \sup _\alpha \left(\sum _{k=1}^n p_k\right)a_\alpha \left(\sum _{k=1}^n p_k\right)\in \left(\sum _{k=1}^n p_k\right) A\left(\sum _{k=1}^n p_k\right).$$

Hence, $\{a_{\xi \eta }\}=\{\sup _\alpha p_\xi a_\alpha p_\xi \}\cup \{p_\xi (\sup _\alpha (p_\xi +p_\eta ) a_\alpha (p_\xi +p_\eta ))p_\eta \}_{\xi \ne \eta }$ is an element of the order unit space $\sum\nolimits _{\xi ,\eta }^\oplus p_\xi Ap_\eta$. It can be checked straightforwardly using the definition of the order in $\sum\nolimits _{\xi ,\eta }^\oplus p_\xi Ap_\eta$ that the element $\{a_{\xi \eta }\}$ is the LUB of the net $(a_\alpha )$. Since the net $(a_\alpha )$ was chosen arbitrarily we obtain the order unit space $\sum\nolimits _{\xi ,\eta }^\oplus p_\xi Ap_\eta$ is monotone compete. $\square$

### **Theorem 4**

Let A be a monotone complete C*-algebra on a Hilbert space H, $\{p_\xi \}$ *be an infinite orthogonal family of projections in A with the LUB 1 in B(H). Suppose that projections in* $\{p_\xi \}$ *are pairwise equivalent and A is not a von Neumann algebra. Then* $A\ne \sum\nolimits _{\xi ,\eta }^\oplus p_\xi Ap_\eta$ (*i.e.* ${\mathcal{A}}:=\{\{p_\xi ap_\eta \}: a\in A\}\ne \sum\nolimits _{\xi ,\eta }^\oplus p_\xi Ap_\eta$).

### *Proof*

By the condition there exists a bounded monotone increasing net $(a_\alpha )$ of elements in *A*, the LUB $\sup _{A} a_\alpha$ in *A* and the LUB $\sup _{\sum\nolimits _{\xi \eta }^\oplus p_\xi B(H)p_\eta } a_\alpha$ in $\sum\nolimits _{\xi \eta }^\oplus p_\xi B(H)p_\eta$ of which are distinct. Otherwise *A* is a von Neumann algebra.

By the definition of the order in $\sum\nolimits _{\xi \eta }^\oplus p_\xi B(H)p_\eta$ there exists a projection $p\in \{p_\xi \}$ such that the LUB $\sup _{pAp} pa_\alpha p$ in *pAp* and the LUB $\sup _{pB(H)p} p a_\alpha p$ in *pB*(*H*)*p* of the bounded monotone increasing net $(pa_\alpha p)$ of elements in *pAp* are different. Indeed, let $a=\sup _A a_\alpha$, $b=\sup _{\sum\nolimits _{\xi \eta }^\oplus p_\xi B(H)p_\eta } a_\alpha$. Since $A\subseteq \sum\nolimits _{\xi \eta }^\oplus p_\xi B(H)p_\eta$, we have $b\le a$ and $0\le a-b$. Hence, if $p_\xi (a-b) p_\xi =0$ for all $\xi$, then $p_\xi (a-b)=(a-b)p_\xi =0$. Therefore by Lemma 2 in Arzikulov (2012) $a-b=0$, i.e. $a=b$. Hence *pAp* is not a von Neumann algebra.

There exists an infinite orthogonal family $\{e_i\}$ of projections in *pAp*, the LUB $\sup _{pAp} e_i$ in *pAp* and the LUB $\sup _{pB(H)p} e_i$ in *pB*(*H*)*p* of which are different. Otherwise *pAp* is a von Neumann algebra.

Indeed, every maximal commutative subalgebra $A_o$ of *pAp* is monotone complete. For each normal positive linear functional $\rho \in B(H)$ and for each infinite orthogonal family $\{q_i\}$ of projections in $A_o$ $\rho (\sup _i q_i)=\sum\nolimits _i \rho (q_i)$, where $\sup _i q_i$ is the LUB of the set $\{q_i\}$ in $A_o$. Hence by the theorem on extension of a $\sigma$-additive measure to a normal linear functional $\rho \vert _{A_o}$ is a normal functional on $A_o$. Hence $A_o$ is a commutative von Neumann algebra. At the same time the maximal commutative subalgebra $A_o$ of the algebra *pAp* is chosen arbitrarily. Therefore by Pedersen (1968) *pAp* is a von Neumann algebra. What is impossible.

Let $\{x_{\xi \eta }\}$ be a set of isometries in *A* such that $p_\xi =x_{\xi \eta }x_{\xi \eta }^*$, $p_\eta =x_{\xi \eta }^*x_{\xi \eta }$ for all $\xi$, $\eta$ and $p_1=p$. Let $\{x_{1\xi }\}$ be the subset of the set $\{x_{\xi \eta }\}$ such that $p_1=x_{1\xi }x_{1\xi }^*$, $p_\xi =x_{1\xi }^*x_{1\xi }$ for all $\xi$. Without loss of generality we assume the set of indices *i* for $\{e_i\}$ is a subset of the set of indices $\xi$ for $\{p_\xi \}$. Let $\{e_ix_{1i}\}$ be the infinite dimensional matrix such that the components, which are not presented, are zeros and $\{x_{1i}^*e_i^*\}$ be a similar matrix. Then $\{x_{1i}^*e_i^*\}$ is the conjugation of $\{x_{1i}^*e_i^*\}$ and $\sum\nolimits _i e_ix_{1i}x_{1i}^*e_i^*=\sum\nolimits _i e_ip_1e_i^*=\sum\nolimits _i e_ie_i^*=\sum\nolimits _i e_i\le \sup _{pAp} e_i$. Therefore $\{a_{\xi \eta }\}\in \sum\nolimits _{\xi ,\eta }^\oplus p_\xi Ap_\eta$. Then $\{a_{\xi \eta }^*\}\in \sum\nolimits _{\xi ,\eta }^\oplus p_\xi Ap_\eta$. Therefore, if $\{a_{\xi \eta }\}\in A$ (i.e. in ${\mathcal{A}}:=\{\{p_\xi ap_\eta \}: a\in A\}$), then the product $\{a_{\xi \eta }\}\cdot \{a_{\xi \eta }^*\}$ in $\sum\nolimits _{ij}^\oplus p_i B(H)p_j$ belongs to $\sum\nolimits _{\xi ,\eta }^\oplus p_\xi Ap_\eta$. In this case the infinite dimensional matrix $\{a_{\xi \eta }\}\cdot \{a_{\xi \eta }^*\}$ contains the component $\sum\nolimits _i e_ix_{1i}\cdot x_{1i}^*e_i^*$ such that $\sum\nolimits _i e_ix_{1i}\cdot x_{1i}^*e_i^*=p_1(\sum\nolimits _i e_ix_{1i}\cdot x_{1i}^*e_i^*)p_1$. Hence $p_1(\{a_{\xi \eta }\}\cdot \{a_{\xi \eta }^*\})p_1=\sum\nolimits _i e_ix_{1i}\cdot x_{1i}^*e_i^*$ and $\sum\nolimits _i e_ix_{1i}\cdot x_{1i}^*e_i^*\in p_1 (\sum\nolimits _{\xi ,\eta }^\oplus p_\xi Ap_\eta )p_1=p_1Ap_1$. Since $\sum\nolimits _i e_ix_{1i}\cdot x_{1i}^*e_i^*=\sum\nolimits _i e_ip_1e_i^*=\sum\nolimits _i e_ie_i^*=\sum\nolimits _i e_i$ we obtain $\sum\nolimits _i e_i\in p_1Ap_1$, i.e. $\sup _{pB(H)p} e_i\in p_1Ap_1$. The last statement is a contradiction. Therefore $\{a_{\xi \eta }\}\notin A$. Hence $A\ne \sum\nolimits _{\xi ,\eta }^\oplus p_\xi Ap_\eta$, i.e. ${\mathcal{A}}:=\{\{p_\xi ap_\eta \}: a\in A\}\ne \sum\nolimits _{\xi ,\eta }^\oplus p_\xi Ap_\eta$. $\square$

The following corollary follows from Theorem 4 and it’s proof.

### **Corollary 1**

Let A be a C*-algebra on a Hilbert space H, $\{p_\xi \}$ *be an infinite orthogonal family of projections in A with the LUB 1 in B(H). Then the following statements are valid:*

1. *suppose that the order unit space* $\sum\nolimits _{\xi ,\eta }^\oplus p_\xi Ap_\eta$ *is monotone complete and there exists a bounded monotone increasing net* $(a_\alpha )$ *of elements in* $\sum\nolimits _{\xi ,\eta }^\oplus p_\xi Ap_\eta$, *the LUB* $\sup _{\sum\nolimits _{\xi ,\eta }^\oplus p_\xi Ap_\eta } a_\alpha$ *in* $\sum\nolimits _{\xi ,\eta }^\oplus p_\xi Ap_\eta$ *and the LUB* $\sup _{\sum\nolimits _{\xi \eta }^\oplus p_\xi B(H)p_\eta } a_\alpha$ *in* $\sum\nolimits _{\xi \eta }^\oplus p_\xi B(H)p_\eta$ *of which are distinct. Then the vector space* $\sum\nolimits _{\xi ,\eta }^\oplus p_\xi Ap_\eta$ *is not closed with respect to the multiplication of* $\sum\nolimits _{\xi ,\eta }^\oplus p_\xi B(H)p_\eta$,
2. *if* $\sum\nolimits _{\xi ,\eta }^\oplus p_\xi Ap_\eta$ is a C*-algebra then this algebra is a von Neumann algebra.

## Application

Let *n* be an infinite cardinal number, $\Xi$ be a set of indices of cardinality *n*. Let $\{e_{ij}\}$ be the set of matrix units such that $e_{ij}$ is a $n\times n$-dimensional matrix, i.e. $e_{ij}=(a_{\alpha \beta })_{\alpha \beta \i \Xi }$, (*i*, *j*)-th component of which is 1, i.e. $a_{ij}=1$, and the other components are zeros. Let *X* be a hyperstonean compact, *C*(*X*) be the commutative algebra of all complex-valued continuous functions on the compact *X* and

$$\begin{aligned} {\mathcal{M}}= &\, \left\{\{\lambda ^{ij}(x)e_{ij}\}_{ij\in \Xi }: (\forall ij\,\,\, \lambda ^{ij}(x)\in C(X))\right.\\ &\left.(\exists K\in {\mathbb{R}})(\forall m\in N)(\forall \{e_{kl}\}_{kl=1}^m\subseteq \{e_{ij}\})\left\Vert \sum _{kl=1\ldots m}\lambda ^{kl}(x)e_{kl}\right\Vert \le K\right\}, \end{aligned}$$

where $\Vert \sum\nolimits _{kl=1\ldots m}\lambda ^{kl}(x)e_{kl}\Vert \le K$ means $(\forall x_o\in X) \Vert \sum\nolimits _{kl=1\ldots m}\lambda ^{kl}(x_o)e_{kl}\Vert \le K$. The set ${\mathcal{M}}$ is a vector space with point-wise algebraic operations. The map $\Vert \,\,\, \Vert : {\mathcal{M}}\rightarrow {\mathbb{R}}_+$ defined as

$$ \Vert a \Vert = \sup _{\{e_{kl}\}_{kl=1}^n\subseteq \{e_{ij}\}}\left\Vert \sum _{kl=1}^n \lambda ^{kl}(x)e_{kl}\right\Vert ,$$

is a norm on the vector space ${\mathcal{M}}$, where $a\in {\mathcal{M}}$ and $a=\{\lambda ^{ij}(x)e_{ij}\}$.

In ${\mathcal{M}}$ we introduce an associative multiplication as follows: if $x=\{\lambda ^{ij}(x)e_{ij}\}$, $y=\{\mu ^{ij}(x)e_{ij}\}$ are elements of *V* then $xy=\{\sum\nolimits _\xi \lambda ^{i\xi }(x)\mu ^{\xi j}(x)e_{ij}\}$. With respect to this multiplication ${\mathcal{M}}$ becomes an associative algebra.

### **Theorem 5**

${\mathcal{M}}$ *is a von Neumann algebra of type I* $_n$ *and* ${\mathcal{M}}=C(X)\otimes M_n({\mathbb{C}})$.

### *Proof*

It is known that the vector space $C(X,M_n({\mathbb{C}}))$ of continuous matrix-valued maps on the compact *X* is a C*-algebra. Let $A=C(X,M_n({\mathbb{C}}))$ and $e_i$ be a $e_{ii}$-valued constant map on *X*, i.e. $e_i$ is a projection in *A*. A C*-algebra *A* is embedded in *B*(*H*) for some Hilbert space *H* such that $\{e_i\}$ is an orthogonal family of projections with $\sup _i e_i=1$ in *B*(*H*). Then $\sum\nolimits _{ij}^\oplus e_iAe_j={\mathcal{M}}$ and $\sum\nolimits _{ij}^\oplus e_iAe_j$ is embedded in *B*(*H*). We have $e_iAe_i=C(X)e_i$ for each *i*, i.e. $e_iAe_i$ is weakly closed in *B*(*H*) for each *i*. Hence by Theorem 3 the image of ${\mathcal{M}}$ in *B*(*H*) is a von Neumann algebra. Hence ${\mathcal{M}}$ is a von Neumann algebra. Note that $\{e_i\}$ is a maximal orthogonal family of abelian projections with the central support 1. Hence ${\mathcal{M}}$ is a von Neumann algebra of type I$_n$. Moreover the center $Z({\mathcal{M}})$ of ${\mathcal{M}}$ is isomorphic to *C*(*X*) and ${\mathcal{M}}=C(X)\otimes M_n({\mathbb{C}})$. The proof is complete. $\square$

## Conclusions

We conclude that a C*-algebra coincides with its IOD if and only if this C*-algebra is weakly closed. If an IOD of a C*-algebra is weakly closed, then this IOD is a von Neumann algebra. The construction of IOD is useful in investigating of operators and C*-algebras. The norm of an infinite dimensional matrix is equal to the supremum of norms of all finite dimensional main diagonal submatrices of this matrix and an infinite dimensional matrix is positive if and only if all finite dimensional main diagonal submatrices of this matrix are positive. Also we conclude that our ideas explained in the present paper may be applied to linear operators, matrices and algebraic structures as Jordan algebras and Lie algebras.

## Abbreviations

LUB: least upper bound
IOD: infinite order decomposition
